# Cell-Autonomous Function of Runx1 Transcriptionally Regulates Mouse Megakaryocytic Maturation

**DOI:** 10.1371/journal.pone.0064248

**Published:** 2013-05-23

**Authors:** Niv Pencovich, Ram Jaschek, Joseph Dicken, Ayelet Amit, Joseph Lotem, Amos Tanay, Yoram Groner

**Affiliations:** 1 Department of Molecular Genetics, The Weizmann Institute of Science, Rehovot, Israel; 2 Department of Computer Science and Applied Mathematics, The Weizmann Institute of Science, Rehovot, Israel; Georg Speyer Haus, Germany

## Abstract

RUNX1 transcription factor (TF) is a key regulator of megakaryocytic development and when mutated is associated with familial platelet disorder and predisposition to acute myeloid leukemia (FPD-AML). We used mice lacking Runx1 specifically in megakaryocytes (MK) to characterized Runx1-mediated transcriptional program during advanced stages of MK differentiation. Gene expression and chromatin-immunoprecipitation-sequencing (ChIP-seq) of Runx1 and p300 identified functional Runx1 bound MK enhancers. Runx1/p300 co-bound regions showed significant enrichment in genes important for MK and platelet homeostasis. Runx1 occupied genomic regions were highly enriched in RUNX and ETS motifs and to a lesser extent in GATA motif. Megakaryocytic specificity of Runx1/P300 bound enhancers was validated by transfection mutagenesis and Runx1/P300 co-bound regions of two key megakaryocytic genes *Nfe2* and *Selp* were tested by *in vivo* transgenesis. The data provides the first example of genome wide Runx1/p300 occupancy in maturating primary FL-MK, unravel the Runx1-regulated program controlling MK maturation *in vivo* and identify a subset of its *bona fide* regulated genes. It advances our understanding of the molecular events that upon RUNX1mutations in human lead to the predisposition to familial platelet disorders and FPD-AML.

## Introduction

The RUNX1 transcription factor (TF) is a key gene expression regulator in embryonic and adult hematopoiesis [1]. *RUNX1* resides on human chromosome 21 and is often involved in leukemia associated chromosomal translocations including 8;21 acute myeloid leukemia (AML) and 12;21 acute lymphoblastic leukemia (ALL) [2]. Cases of normal karyotype AML [3], [4] frequently involve point mutations in *RUNX1*. RUNX1 plays a key role in the development of MK [5], [6], the polyploid precursors of blood platelets, which are crucial mediators of blood clotting and homeostasis. Over-expression of RUNX1 in myeloid cell lines induces megakaryocytic differentiation [6], [7], while knockdown impairs megakaryopoiesis [8]. Moreover, Runx1 deficiency in adult bone marrow attenuated megakaryocytic maturation and reduced blood platelet numbers (thrombocytopenia) [5]. Haploinsufficiency of RUNX1, due to heterozygous loss-of-function mutations, is associated with familial platelet disorder and predisposition to acute myeloid leukemia (FPD-AML) [9], [10]. Of potential relationship, patients with trisomy 21 (Down syndrome) have a 500 fold-increased risk of developing acute megakaryoblastic leukemia (DS-AMKL/AML-M7) relative to normal individuals [11]. While the importance of RUNX1 in megakaryopoiesis is well-established [5], [12], [13] information about its role in driving the regulatory program of MK maturation and platelet formation in the *in vivo* milieu is lacking, as is the information about RUNX1-direct target genes during the advanced stages of megakaryocytic differentiation.

The murine platelet factor 4 (Pf4) is a member of the CXC chemokine family. Pf4, also known as CXCL4, is released from the alpha granules of activated platelets and is involved in platelet aggregation [14]. Ravid et al [15] have demonstrated that of all circulating blood cells in mice only platelets express Pf4. In the present work we used a transgenic mouse produced by Tiedt et al [16] in which Cre expression is mediated by a modified BAC spanning *Pf4* regulatory region resulting in expression of Cre exclusively in the megakaryocytic lineage [16].

Using MK cell lines we have recently demonstrated that RUNX1 regulates numerous target genes during megakaryocytic differentiation through sequential cooperation with GATA1, AP-1 and ETS TFs [8]. Here we used mice lacking Runx1 in MK to study the Runx1-mediated transcriptional program during normal MK cell maturation. Specific inactivation of Runx1 in maturating MK resulted in aberrant maturation and thrombocytopenia, as was also described by others upon abrogation of Runx1 activity in adult hematopoietic progenitor cells [5]. Genome wide occupancy profile of Runx1 was combined with Runx1-mediated gene expression analysis. The integrative genome occupancy and transcriptome analysis revealed a broad repertoire of Runx1 responsive genes. A subset of Runx1 bound regulatory elements was singled out by ChIP-seq of p300, an enhancer-associated co-activator previously shown to identify functional enhancers *in vivo* [17]. Enhancer functionality was validated by transfection mutagenesis, and p300/Runx1 co-bound regions upstream of two key megakaryocytic genes *Nfe2* and *Selp* [18], [19] were tested by *in vivo* transgenesis. The data provide the first genome wide analysis of Runx1 target genes in maturating primary megakaryocytes and advance our understanding on the regulatory program controlling MK maturation, platelet production and function *in vivo*. It furnishes important insights into the molecular events that lead to development of megakaryocytic pathologies upon compromised RUNX1 activity.

## Methods

### Generation of mice lacking Runx1 in maturating MK and phenotypic evaluations

Runx1*^F^* ^/*F*^/Pf4-Cre mice were created by crossing Runx1*^L^* ^/*L*^ mice [4] with Pf4-Cre transgenic mice [16]. Excision of *Runx1* exon 4 in fetal liver MK (FL-MK) was assessed by Southern blotting using primers listed in Table S2 and loss of Runx1 RNA and protein were evaluated by RT-qPCR and Western blotting, respectively. For colony forming assay, FLs of E14.5 embryos were dissected as previously described [20]. Cells were plated in Methocult methylcellulose medium (Stemcell Technologies) supplemented with mouse thrombopoietin (TPO, Sigma-Aldrich Israel 50 ng ml^−1^) and cultured for 7 days prior to colony count. Complete blood count was performed on ∼200 µl blood samples, obtained by bleeding the retro-orbital sinus, using the SYSMEX K-21 blood analyzer. Ethics Statement: All experiments involving mice were approved by the Weizmann Institute Institutional Animal Care and Use Committee (IACUC permit number.06121110-1).

### Primary mouse FL megakaryocytes

Murine FL derived mature MK were produced essentially as previously described [20]. Briefly, FL cells were derived from E14.5 embryos and cultured in DMEM medium supplemented with 10% FBS (Gibco, US), 2 mM L-glutamine and penicillin/streptomycin and TPO (50 ng ml^−1^) at 37°C and 10% CO_2_. After 7 days in culture mature MK were isolated by two sequential 2%–4% BSA gradients. FACS analysis was used to confirm over 80% purity of maturating megakaryocytes.

### Chromatin Immunoprecipitation-sequencing (ChIP-seq) and data analysis

ChIP was performed essentially as described [21]. Briefly, for each ChIP assay ∼70 E14.5 FLs were processed and chromatin from ∼5×10^7^ mature megakaryocytes was fragmented to an average size of ∼200 bp by 20 cycles of sonication (30sec each) in 15 ml tubes using the Bioruptor UCD-200 sonicator (Diagenod, US). The fragmented chromatin was immunoprecipitated using 60 µl of in house anti-Runx1 antibodies [22] or 400 µl of anti-p300 (C-20: sc-585, Santa Cruz Biotechnology). Rabbit pre-immune serum was used as ChIP control. DNA was purified using QIAquick MinElute columns (QIAGEN, US) and sequenced using Illumina HiSeq according to manufacturer instructions. Two biological repeats were conducted for each ChIP experiment and separately sequenced. For ChIP-seq analysis, Illumina sequencing short reads (36 bp) were aligned to the mouse genome (mm9) using the Eland program (Illumina). Multiple reads were discriminated, and coverage profile generated by elongating reads to 200 bp according to mapped strand. Coverage profile was analyzed in bins of 50 bp unless otherwise noted and the distribution of coverage per bin was estimated. We used percentile values from this distribution when thresholding was required as described below. Sequence analysis was performed using Runx1 and p300 bound loci (top 0.1 percentile) that were filtered to remove non-immune serum loci that displayed significant ChIP-seq coverage (top 1.0 percentile). ChIP-seq data will be deposited in a publically available database upon acceptance for publication.

### Microarray processing and analysis

RNA and was isolated using the EZ-RNA (Biological Industries, Beit Haemek Israel), according to manufacturer instructions. Purified RNA was reverse-transcribed, amplified and labeled with Affymetrix GeneChip whole transcript sense target labeling kit. Labeled cDNA from mature MK^Runx1−/−^ and MK^Runx1*L*/*L*^ was analyzed using Affymetrix mouse gene ST 1.0 microarrays, according to manufacturer instructions. Microarrays were scanned by GeneChip scanner 3000 7 G and the data was normalized using RMA. Microarray data will be deposited in a publically available database upon acceptance for publication.

### Regulatory element-reporter constructs and RT-qPCR

Genomic DNA fragments of *Nfe2, Selp, Pde3a* and *Lrrc32* regulatory elements were generated by PCR amplification of regions spanning the following coordinates: Nfe2: chr-6:103088686–103089226; Selp: chr-1:166065335–166066395; Pde3a: chr-6:141243636–141244353; Lrrc32: chr-7:105682347–105683400. Fragments were then cloned into the Bgl2 site of TK-promoter PGL4.73 vector (Promega, US). TFSEARCH (www.cbrc.jp/research/db/TFSEARCH.htm) identified RUNX motifs were mutated by overlap PCR (OE-PCR) using the following primers: Nfe2: ACCGCA to ATTAGA, Selp: ACCACA to ATTCCA, Pde3a: ACCACA to AGTCTA (two adjacent sites), Lrrc32: TGTGGT to TCACTT. Nfe2 and Selp regulatory elements were further cloned into the Hind3 and Sph1 sites of Hsp68-GFP reporter plasmid (Bee et al, 2009). qPCR assays were performed using light cycler 480 (Roche, US) with 480 SYBR Green I Master (Roche,US). Table S2 lists the primers used for cloning of the TK promoter into Pgl4.73 vector, for genomic DNA fragments amplification, for site-specific mutagenesis and PCR.

### Definition of RUNX1 target genes

Gene expression analyses in MK^Runx1−/−^ and MK^Runx1*L*/*L*^ were compared and fold expression difference was defined as the ‘Runx1 Effect’. For computation of global statistics, genes were considered transcriptionally activated or repressed by Runx1 when displaying a minimal Runx1-dependent effect of 1.5-fold or higher. To derive the relationship between Runx1 occupancy and change in gene expression we calculated the density of Runx1 bound loci as a function of the distance from genes transcriptional start site (TSS) averaged across activated, repressed or non-responsive genes, thereby quantifying the enrichment of potential regulatory sites in these groups.

### Motifs finding and binding energy

To characterize the sequence preferences of Runx1 bound sites, we searched *de-novo* for discriminating position weight matrices (PWM) and computed their match to the sequence using their *approximated binding energy* as defined in Pencovich et al 2011 [8]. Briefly, the approximated binding energy is the integrating contributions from all positions in a given region, weighted according to the similarity of their sequence to PWM model preferences. Since the model is multiplicative, a single consensus sequence will dominate the binding energy of a region. On the other hand, several weaker sequence motifs may still combine to form a high or medium binding potential. *De-novo* motif finding was performed as previously described [8], using regions flanking Runx1 and/or p300 peaks (500 bp) compared to background samples from regions 1 kb upstream or downstream these peaks. The entire dataset was uploaded to the Gene Expression Omnibus Database (Accession Number GSE45374) at NCBI for public access (http://www.ncbi.nlm.nih.gov/geo/query/acc.cgi?acc=GSE45374).

## Results

### Runx1 is essential for maturation of megakaryocytes

**Figure 1:**
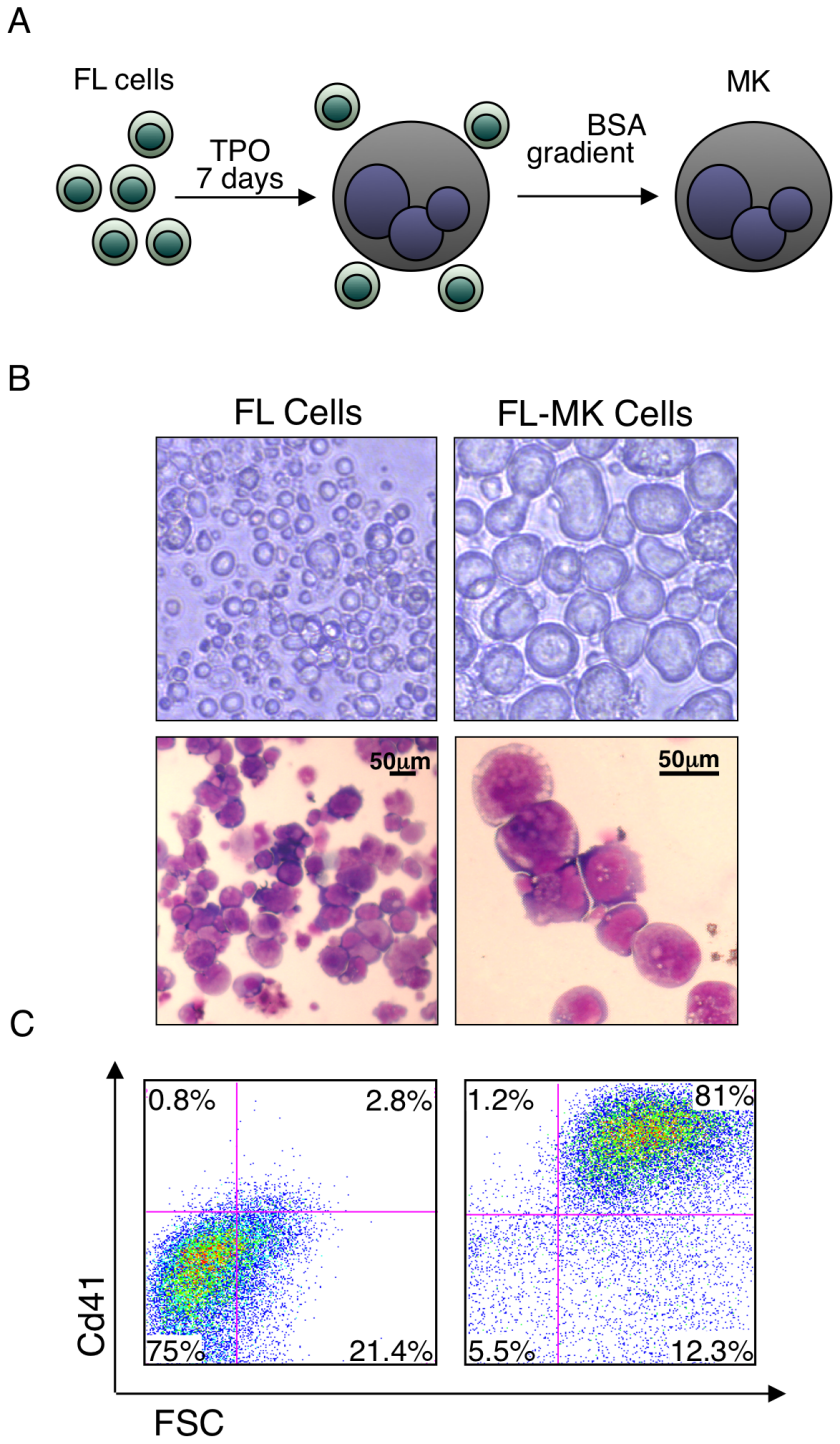
Characterization of FL derived maturating megakaryocytes. (A) Schematic representation of timeline for producing of FL-MK. E14.5 FL single cells were cultured with TPO 50 ng ml^−1^ for 7 days prior to 2%–4% BSA gradient for MK separation. (B) Upper panels; light microscopy images of upper (left) or lower (right) part of a BSA gradient fractionation of cultured 7-days FL cells. Lower panels; microscopic images of May-Grünwald-Giemsa stained cultured FL cells derived from the lower part of a BSA gradient. Upper panel images were produced with an Olympus IX71 microscope with 10×/0.3 (left) and 40×/0.6 (right) objective lenses. Lower panel images were produced with a Nikon Eclipse 800 microscope equipped with a 40×/1.25 objective lens (left) or 100×/1.25 lens (right) and numeric aperture oil objective lenses and Nikon DXM1200 digital camera with Nikon ACT-1 2.63 software.(C) Flow cytometry analysis of the FL cells shown in (B) stained with Cd41-PE antibody. The culture contains at least 80% enrichment of large size Cd41 positive MK. Shown are representative images of at least 3 biological repeats with similar results.

Murine FL cells derived from E14.5 embryos were cultured for 7 days with thrombopoietin (TPO). Cultured primary cells were gently separated by BSA gradients (Figure 1A) to minimize damage to the fragile mature large size MK. Separated cells were highly enriched for large, CD41^+^ MK (Figure 1B and C). However, due to lack of synchrony, variations in size and morphology that reflected differences in MK maturation stage were noted (Figure 1B). Using this procedure we processed 70 E14.5 FL and collected ∼10^7^ mature MK that were subsequently used for gene expression and ChIP-seq analysis.

**Figure 2:**
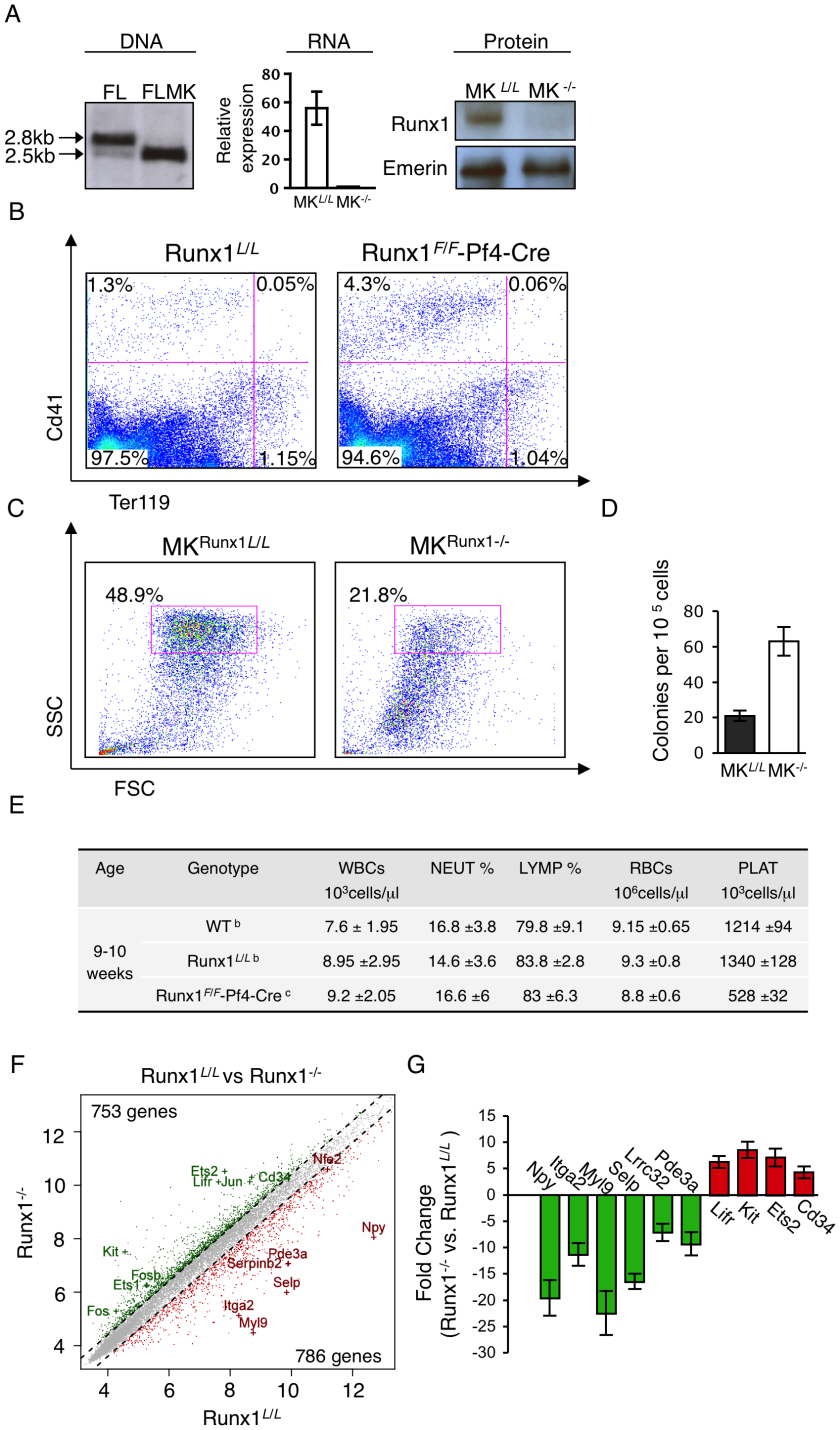
Loss of Runx1 impairs FL-MK maturation. (A) Left panel; Southern blot analysis of *BamH1* digested E14.5 FL cell DNA from Runx1*^F^* ^/*F*^-Pf4-Cre mice (FL) and mature purified FL derived MK^Runx1−/−^ (FLMK). Middle panel; RT-qPCR analysis of *Runx1* expression in MK^Runx1−/−^ relative to MK^Runx1*F*/*F*^ cells. Data represent the mean ± SD of two independent experiments performed in triplicates. Right panel; Western blot analysis of FL-MK^Runx1*L*/*L*^ (MK*^L^* ^/*L*^) and FL-MK^Runx1−/−^ (MK^−/−^) nuclear extracts using anti-Runx1 antibody. Emerin was used to monitor the amount of loaded protein. (B) FACS analysis of bone marrow (BM) cells derived from Runx1*^L^* ^/*L*^ and Runx1*^F^* ^/*F*^-Pf4-Cre mice. Cells were cultured for 3 days with TPO 50 ng ml^−1^ stained with anti-CD41-PE and anti Ter119-FITC antibodies and analyzed by FACS. (C) FACS analyses of cultured (7 days plus TPO) FL MK^Runx1*L*/L^ and MK^Runx1−/−^ by forward scatter (FSC) Vs side scatter (SSC). (D) Assessment of colony forming unit megakaryocytes (CFU-meg) of E-14.5 FL cells derived from Runx1*^L^* ^/*L*^ or Runx1^F/F^-Pf4-Cre embryos. Data represent the mean ± SD of two independent experiments performed in triplicates. Increased colony numbers in Runx1*^F^* ^/*F*^-Pf4-Cre, relative to Runx1*^L^* ^/*L*^ mice, was statistically significant [*P* = 0.002]. (E) Analysis of hematopoietic indices in blood of WT, Runx1*^L^* ^/*L*^ and Runx1*^F^* ^/*F*^-Pf4-Cre mice. Analysis was carried out using blood samples (n = 5^b^ or 8^c^) obtained by bleeding the orbital sinus: RBCs, red blood cells; WBCs, white blood cells; NEUT, neutrophils; LYMP, lymphocytes; PLAT, platelets.(F) Gene expression alterations in maturating MK lacking Runx1. Genes are plotted based on their expression level (log2 scale) in MK^Runx1−/−^ vs MK^Runx1*L*/*L*^. Genes showing 1.5-fold increase or decrease in expression levels are indicated in green or red, respectively. Examples of up/down-regulated genes known to play role in megakaryocytic differentiation and cell proliferation are indicated. (G) Validated expression levels of several Runx1 responsive genes by RT-qPCR using RNA from either FL-MK^Runx1*L*/*L*^ or FL-MK^Runx1−/−^. The data represent means ± SD of two experiments performed in triplicates.

To assess the role of Runx1 during advanced stages of megakaryopoietic differentiation, we generated megakaryocyte-specific Runx1-deficient mice by crossing *Runx1* loxP-flanked exon 4 (Runx1*^L^* ^/*L*^) mice [4] onto Pf4-Cre transgenic mice [16]. Because Pf4 expression is confined to mature CD41^+^ MK and platelets [23], *Runx1*in these Runx1*^F^* ^/*F*^/Pf4-Cre mice was inactivated specifically in advance stages of MK maturation (Figure 2A). Thus, FL-derived primary MK obtained from Runx1*^F^* ^/*F*^/Pf4-Cre mice (FL-MK^Runx1−/−^) provided unique means for studying Runx1 function explicitly during terminal stages of megakaryocytic maturation.

Phenotypically, Runx1*^F^* ^/*F*^-Pf4-Cre mice displayed megakaryocytic defects similar to those previously noticed upon abrogation of Runx1 activity in hematopoietic progenitor cells [5]. The megakaryocytic lineage of Runx1*^F^* ^/*F*^/Pf4-Cre mice had a higher proliferative capacity, evidenced by increased percentage of Cd41^+^ BM cells compared to Runx1*^L^* ^/*L*^ mice (Figure 2B). On the other hand, the percentage of Ter119^+^ cells, representing the erythrocyte lineage was normal (Figure 2B). MK^Runx1−/−^ cells were smaller, less granular (Figure 2C), showed increased megakaryocytic-colony-forming ability compared to FL-MK^Runx1*L*/*L*^ (Figure 2D) and expressed lower levels of megakaryocytic markers (not shown). Finally, Runx1*^F^* ^/*F*^/Pf4-Cre mice suffer from low platelet counts in peripheral blood (thrombocytopenia) (Figure 2E), consistent with abnormal megakaryocytic maturation. Overall, Runx1*^F^* ^/*F*^/Pf4-Cre mice display a phenotype of aberrant megakaryopoiesis in which megakaryoblastic proliferation is favored over differentiation. These findings underscore the notion that in addition to its function in early megakaryopoiesis, Runx1 also plays a major role during the final stages of megakaryocytic maturation.

### Runx1 is a major gene expression regulator in maturating megakaryocytes

To directly assess the changes in gene expression underlying the contribution of Runx1 to the MK maturation defect manifested in Runx1^F/F^/Pf4-Cre mice, we analyzed FL-MK derived from Runx1*^L^* ^/*L*^ and Runx1*^F^* ^/*F*^/Pf4-Cre mice. Comparison of gene expression in FL-MK^Runx1*L/L*^ Vs. FL-MK^Runx1−/−^ revealed pronounced changes (Figure 2F), underscoring the central role of Runx1 in homeostasis of maturating megakaryocytes. In cells lacking Runx1 the expression of 1539 genes was changed, compared to FL-MK^Runx1*L*/*L*^. Of these Runx1 responding genes 786 and 753 were either down- or up-regulated, respectively (>1.5 fold, FDR<0.05). Many of the responding genes including *Itga2, Itgal, Lrrc32, Mkl, Myl9, Pde3a, Pik3cb/g, Selp and Was* are known to play a role in megakaryopoiesis and in platelet biogenesis and function (Table S1 and Refs therein, and Figure 2F and G), in good agreement with the impaired FL-MK^Runx1−/−^ maturation and the phenotype of Runx1*^F^* ^/*F*^/Pf4-Cre mice (Figure 2B-E). Furthermore, higher expression of known oncogenes such as *Bcl-2, Bcl-3*, *Bcl-6, Ets1, Ets2, Fos, Fosb, Jun, Junb and Kit* was also noted in Runx1^−/−^ cells (Table S1 and Refs therein, and Figure 2F and G), consistent with the increased proliferation of FL-MK^Runx1−/−^ and the predisposition to leukemia in humans with RUNX1 deficiency.

### Genome-wide occupancy of Runx1 and p300 in maturating megakaryocytes

**Figure 3:**
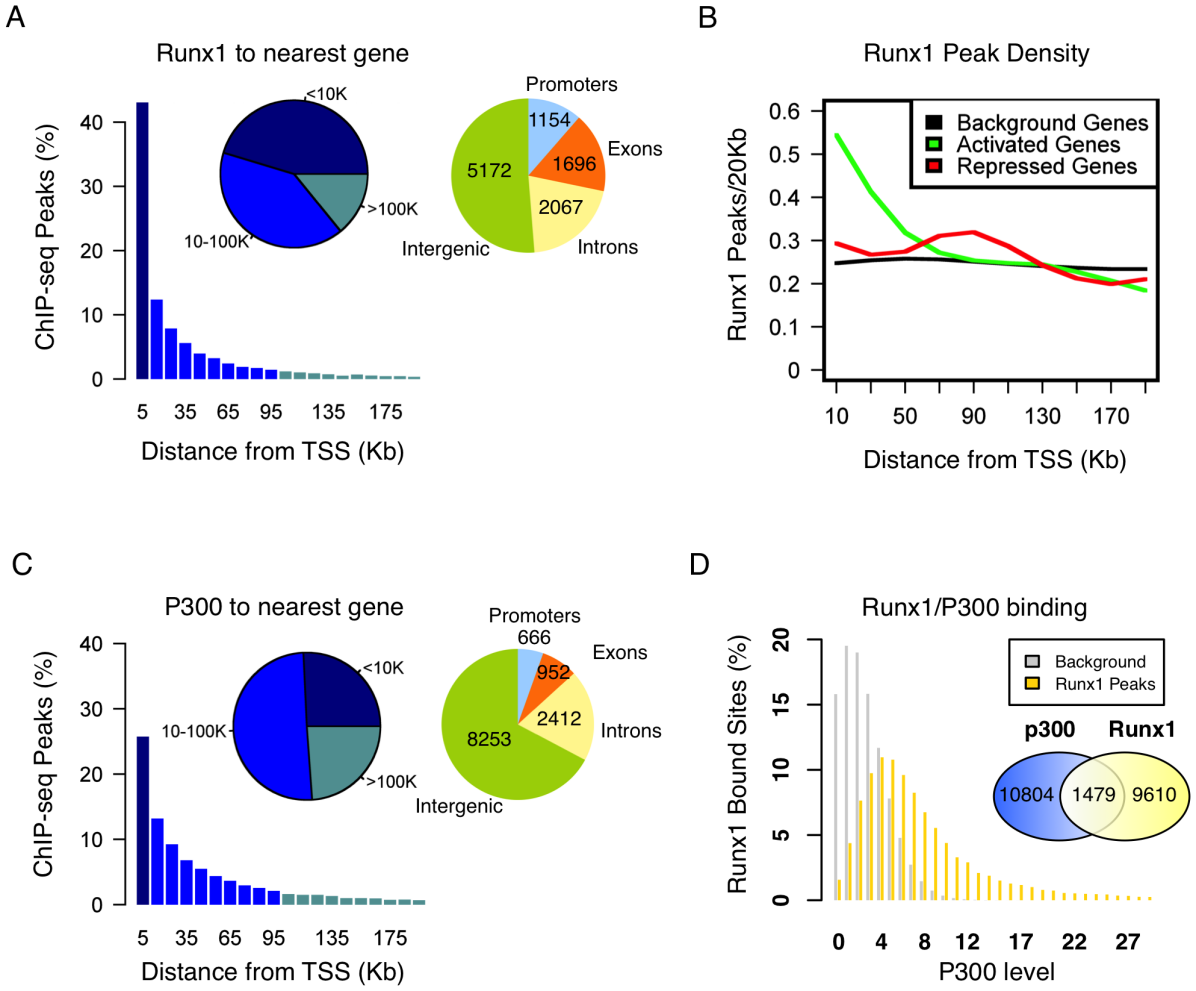
Genome occupancy of Runx1 and p300 in maturating MK. (A) Distribution of Runx1 ChIP-seq peaks relative to gene TSS. Shown are Runx1 peak frequencies relative to the distance from the nearest annotated TSS. Pie charts represent groups of peak-distances from TSS (left) and peak distributions among genomic constituents (right). (B) Enrichment of Runx1 binding in proximity of activated/repressed genes. The density (peaks/20 kb, Y-axis) of Runx1 bound sites is plotted relative to TSSs for Runx1-activated (green) or repressed (red) genes, relative to background genes (black). The enrichment of RUNX1 peaks within 20 kb of activated genes and within 60 kb–100 kb of repressed genes was significant (2.6 fold enrichment within 20 kb from TSS, P<<1e-10, and 1.4 fold enrichment within 60 kb–100 kb from TSS, P<<1e-10). (C) Genome distribution of p300 ChIP-seq peaks. Shown are frequencies of p300 peaks relative to the distance from the nearest annotated TSS. Pie charts represent groups of peak-distances from TSS (left) and peak distributions among genomic constituents (right). (D) Co-binding of Runx1 and p300 in maturating MK. Distribution of p300 ChIP-seq reads at Runx1 bound regions (yellow) and at background regions (gray). Venn diagram summarizing the overlap between Runx1 and p300 bound sites in maturating MK. Runx1 and p300 peaks, assessed from analysis of two independent ChIP-seq experiments for each. Occupied regions were defined as those that are above threshold and lack significant binding in NIS ChIP-seq.

Runx1 ChIP-seq assays were performed using FL-MK^Runx1*L*/*L*^ to obtain a comprehensive view of Runx1 genome occupancy in maturating MK. Peak-calling assessment using a permissive threshold revealed 11,089 Runx1 bound genomic loci. Location analysis of these Runx1 occupied regions relative to the nearest transcription start sites (TSSs) of annotated genes, revealed that ∼55% were placed more than 10 kb away from any TSS, and ∼20% were in “gene deserts” (above 100 kb from the nearest TSS) (Figure 3A). More specifically, ∼12% of bound sites (1154) are located within promoter regions (up to 3 kb upstream to TSS), ∼51% (5172) in intergenic regions, ∼20% (2067) in introns and ∼17% (1696) in exons (Figure 3A). Integrative analysis of Runx1 occupancy with expression data of Runx1 responsive genes, demonstrated that genes activated by Runx1 were significantly enriched for Runx1 occupancy within 20 kb around their TSSs (Figure 3B), whereas among the repressed genes Runx1 binding was enriched at a somewhat more distal region (60 kb to 100 kb around the TSS) (Figure 3B). Collectively, the data indicate that in maturating MK Runx1 regulates its target genes primarily through binding to long-range regulatory elements and suggest a distance related distinction between up- or down-regulated target genes.

**Figure 4:**
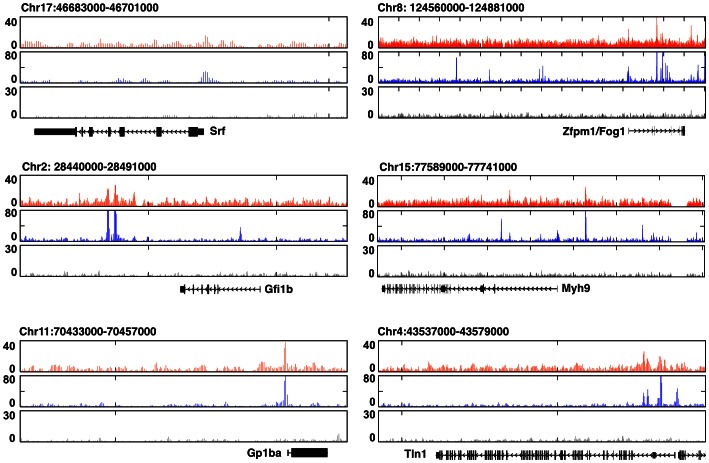
Runx1/p300 co-occupancy of several key MK gene-loci displaying unchanged expression in FL-MK^Runx1−/−^. UCSC browser normalized Runx1 (orange), p300 (blue) and non-immune serum (gray) ChIP-seq traces in maturating FL-MK surrounding the genomic loci of *Srf, Zfpm1, Gfi1b, Myh9, Gp1ba* and *Tln1.*

We next performed p300 ChIP-seq using maturating FL-MK. Peak-calling analysis applying a similar threshold as for Runx1, revealed p300 occupancy at 12,283 genomic regions. Comparison to genomic distribution of Runx1 binding disclosed significantly higher proportion of peaks at regions 10 kb away from TSS (i.e. ∼75% Vs ∼55%), but only ∼5% (Vs ∼12%), and ∼7% (Vs ∼17%) of the peaks were within promoter regions and exons, respectively (Figure 3C). Approximately 25% of p300 bound regions were in “gene deserts”, ∼67% in intergenic regions and ∼20% in introns (Figure 3C). While the majority of the Runx1 bound regions were not marked by p300, 1479 genomic regions were co-bound by both Runx1 and p300 (Figure 3D). Data analysis of Runx1/p300 ChIP-seq results using GREAT [24] indicated that ∼23% of the Runx1-responsive genes were also co-bound by p300 and provided a revealing functional annotation of the p300/Runx1 co-bound gene subset (Table 1). However, the majority of the p300/Runx1 co-bound regions (i.e. ∼73%) corresponded to genes that were not differentially expressed in FL-MK^Runx1−/−^ compared to FL-MK^Runx1*L/L*^. This subset of co-bound non-responsive genes contained several genes with major roles in MK development and platelet biogenesis including the TFs *Srf* [25], [26] and *Zfpm1/Fog1* [27], the transcriptional repressor *Gfi1b* [28], the myosin heavy chain *Myh9* [29], the glycoprotein 1b alpha (*Gp1ba*) [30] and the cytoskeletal protein *Tln*1 [31] (Figure 4). Runx1 may be involved in transcription regulation of these genes during preceding or subsequent stages of MK differentiation and platelet formation.

**Table 1:** GREAT Analysis of Runx1 and p300 co-bound genomic regions show highly significant overrepresentation of genes with important roles in megakaryopoiesis and platelet function.

Enriched term	Raw P-values	FDR Q-values
**Mouse phenotype**		
Abnormal platelet physiology	7.87 e-24	2.41 e-10
Abnormal platelet activation	5.46 e-20	1.49 e-6
Decreased platelet aggregation	2.87 e-18	1.74 e-6
Abnormal megakaryocyte progenitor cell morphology	5.78 e-15	1.13 e-5
Abnormal blood coagulation	1.28 e-16	6.68 e-8
Abnormal megakaryocyte morphology	1.73 e-14	2.32 e-4
Decreased platelet cell number	2.08 e-14	6.03 e-5
Abnormal thrombopoiesis	3.97 e-14	4.63 e-5
Abnormal megakaryocyte differentiation	1.83 e-11	4.1 e-3
**Disease Ontology**		
Blood platelet disease	4.57 e-14	3.2 e-4
Hemorrhagic disease	1.23 e-12	2.72 e-4
Blood coagulation disease	4.29 e-12	3.49 e-4
Thrombocytopenia	1.02 e-6	3.3 e-2

### Sequence specificity of Runx1 occupancy sites

**Figure 5:**
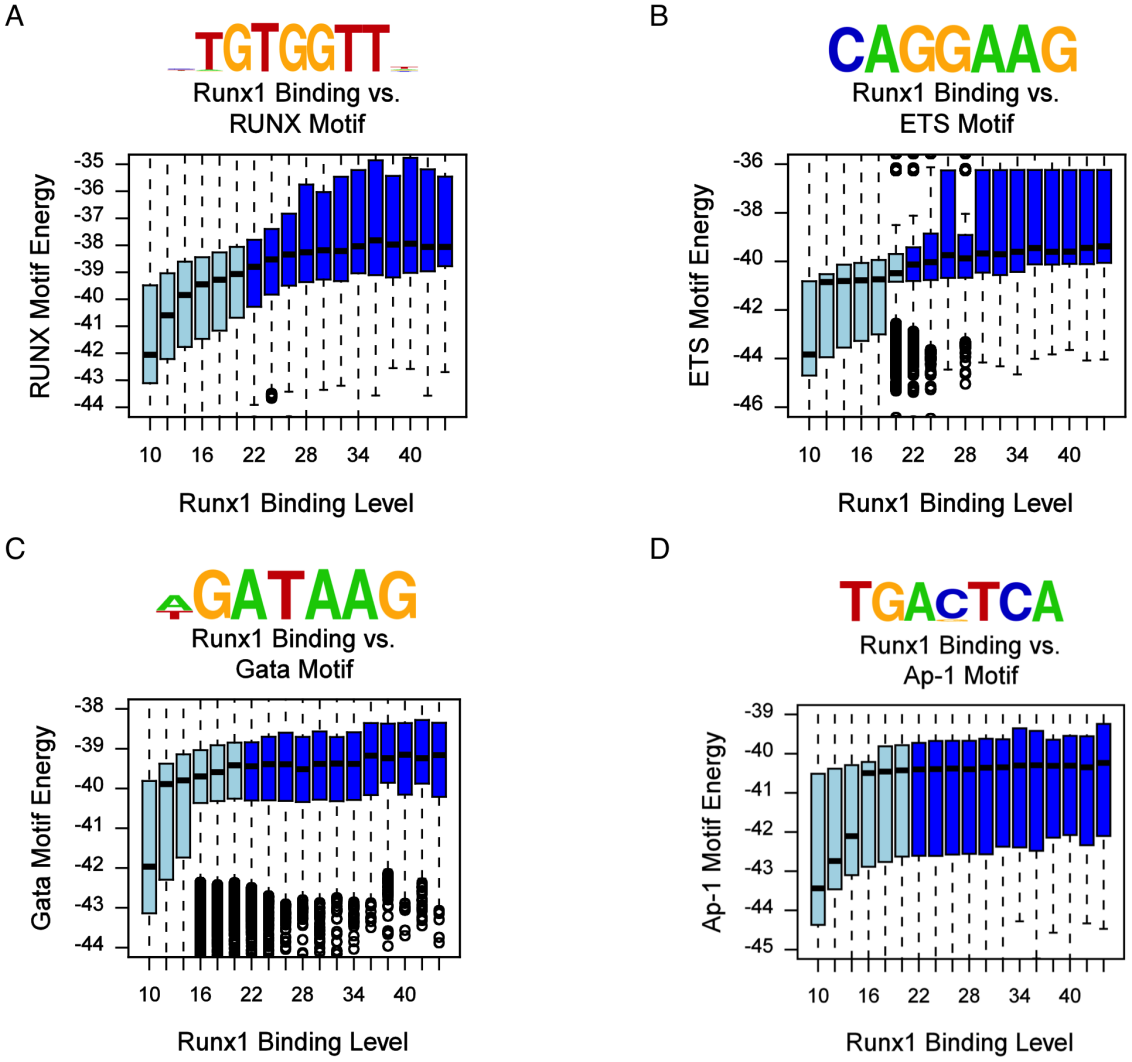
Enrichment of TF motifs in Runx1 bound genomic regions. (A) RUNX motif binding energy is correlated with RUNX1 ChIP-seq occupancy. The indicated RUNX motif was inferred directly from Runx1 occupancy peaks (Methods). Box plots depict the distributions of motif binding energies (Y-axis) in groups of regions with increasing Runx1 ChIP-seq coverage (binding level) (X-axis). The regions that passed the threshold and were thus defined as Runx1 bound are shown in dark blue boxes. The outliers represent values more than 90^th^ percentile. (B) ETS binding motif is correlated with Runx1 ChIP-seq occupancy. An ETS motif was inferred directly from its association with Runx1 bound regions and analysis was conducted as in (A). The correspondence between Runx1 occupancy, GATA and AP-1 motifs binding energies was analyzed as in (A) and is presented in (C) and (D). Box plots depict the distributions of motif binding energies (Y-axis) in groups of regions with increasing Runx1 ChIP-seq readout coverage (X-axis).

To characterize the sequence specificity of Runx1 occupancy sites in maturating MK we searched for DNA sequence motifs within Runx1 bound regions in comparison to background sequences. The most prevalent sequence was the known RUNX motif TGTGGTT, which is identical to the motif found in early differentiating human megakaryocytic cell lines [8]. Further analysis showed that the energy of this motif was strongly correlated with Runx1 binding levels (Figure 5A). Runx1 binding level at occupied regions also correlated with pronounced enrichment of the ETS TF motif CAGGAAG (Figure 5B), while the preponderance of another enriched sequence GATAAG, i.e. the GATA TF motif, was substantially lower (Figure 5C). These findings correspond with the observation that in human megakaryocytic cell lines RUNX1 sequentially cooperate with GATA1/2 and ETS TFs to drive the differentiation program [8]. Interestingly, in sharp contrast to the previously observed co-binding of RUNX1 and AP1 TFs at early megakaryocytic differentiation [8], only poor enrichment in AP1 motif was noted among Runx1 occupied regions of maturating FL-MK (Figure 5D).

### Runx1-mediated transcriptional repression of AP-1 genes

**Figure 6:**
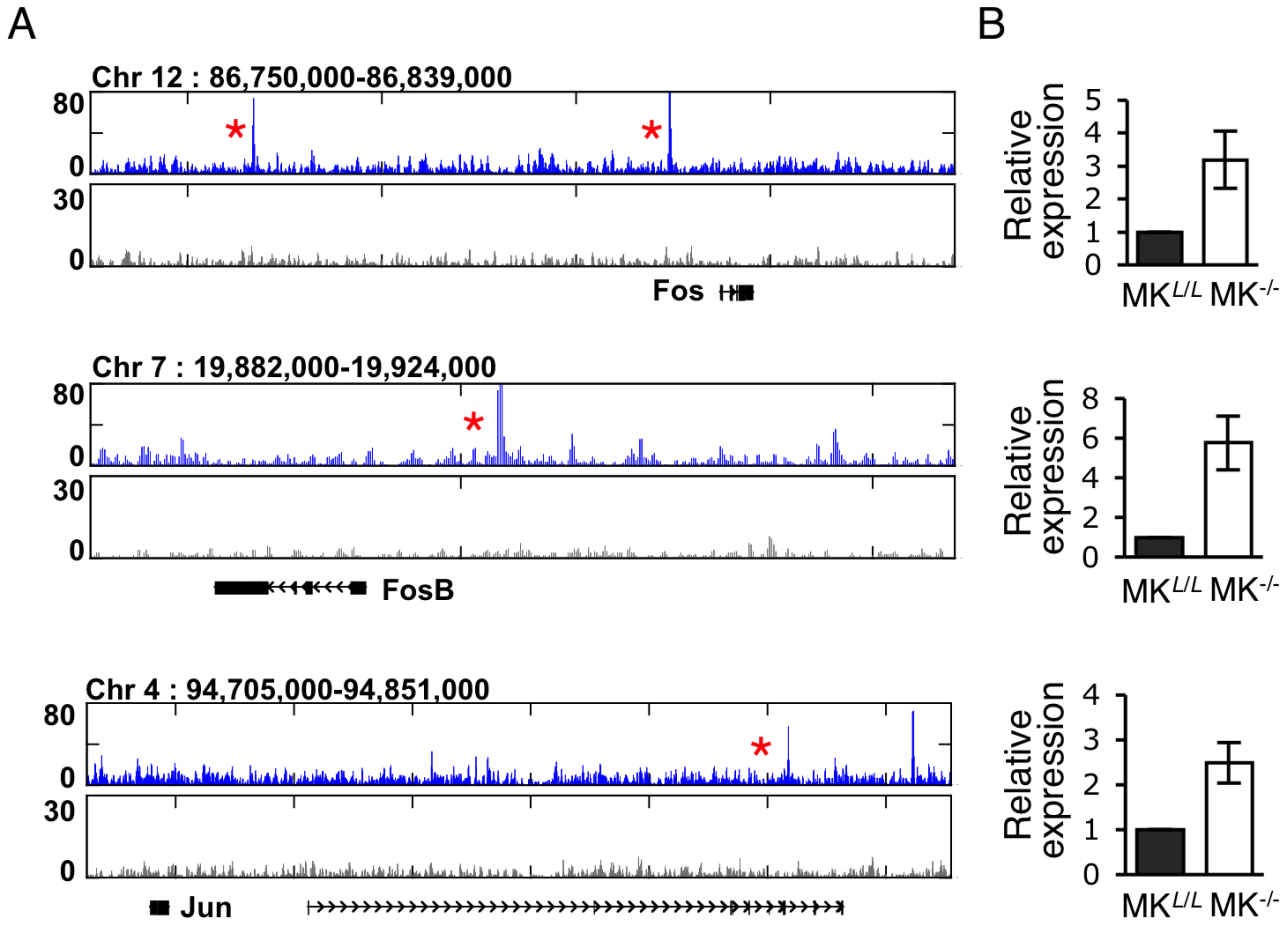
Runx1 down-regulates AP-1 expression in maturating MK. (A) Runx1 binding pattern surrounding genome loci of AP-1 TF. UCSC browser normalized tracks of Runx1 (blue) and non-immune serum (black) ChIP-seq traces surrounding *Fos, Fosb, Jun* and *Junb* loci. Red asterisks mark genomic region that were bound by Runx1 in early stages of MK-cell line differentiation[8]. (B). RT-qPCR analysis of *Fos, Fosb*, *Jun* and *Junb* expression in MK^Runx1*L*/*L*^ (MK*^L^* ^/*L*^) or MK^Runx1−/−^ (MK^−/−^). Data represent the mean ± SD of three independent experiments performed in triplicates. The increased expression of *Fos, Fosb Jun* and *Junb* in MK^Runx1−/−^ (MK^−/−^), relative to MK^Runx1*L*/*L*^ (MK*^L^* ^/*L*^), was significant [*P* = 0.04, 0.004, 0.001 and 0.01, respectively]. PCR primers used are listed in Table S2.

Because upon induction of MK maturation in human cell lines RUNX1 bound and up-regulated the expression of the AP1 TFs *FOS, FOSB* and *JUN*, we assessed the binding of Runx1 to these loci in the maturating FL-MK. ChIP-seq data revealed that *Fos, Fosb* and *Jun* genomic regions, bound by Runx1 in FL-MK (Figure 6A), were by and large similar to those occupied by RUNX1 in early differentiating MK cell lines [8]. However, in maturating MK, the bound Runx1 transcriptionally repressed the AP1 genes as evidenced by the increased expression of *Fos, Fosb* and *Jun* in FL-MK^Runx1−/−^ cells (Figure 6B and Table S1). This finding corresponds with the observation that in MK cell lines, RUNX1 and AP1 are engaged in a regulatory loop [8], manifested in RUNX1-mediated activation at an early stage followed by AP1 gene repression at later stages concomitantly with advancement of the differentiation program.

### Transcriptional activity of Runx1/p300-cobound enhancers

**Figure 7:**
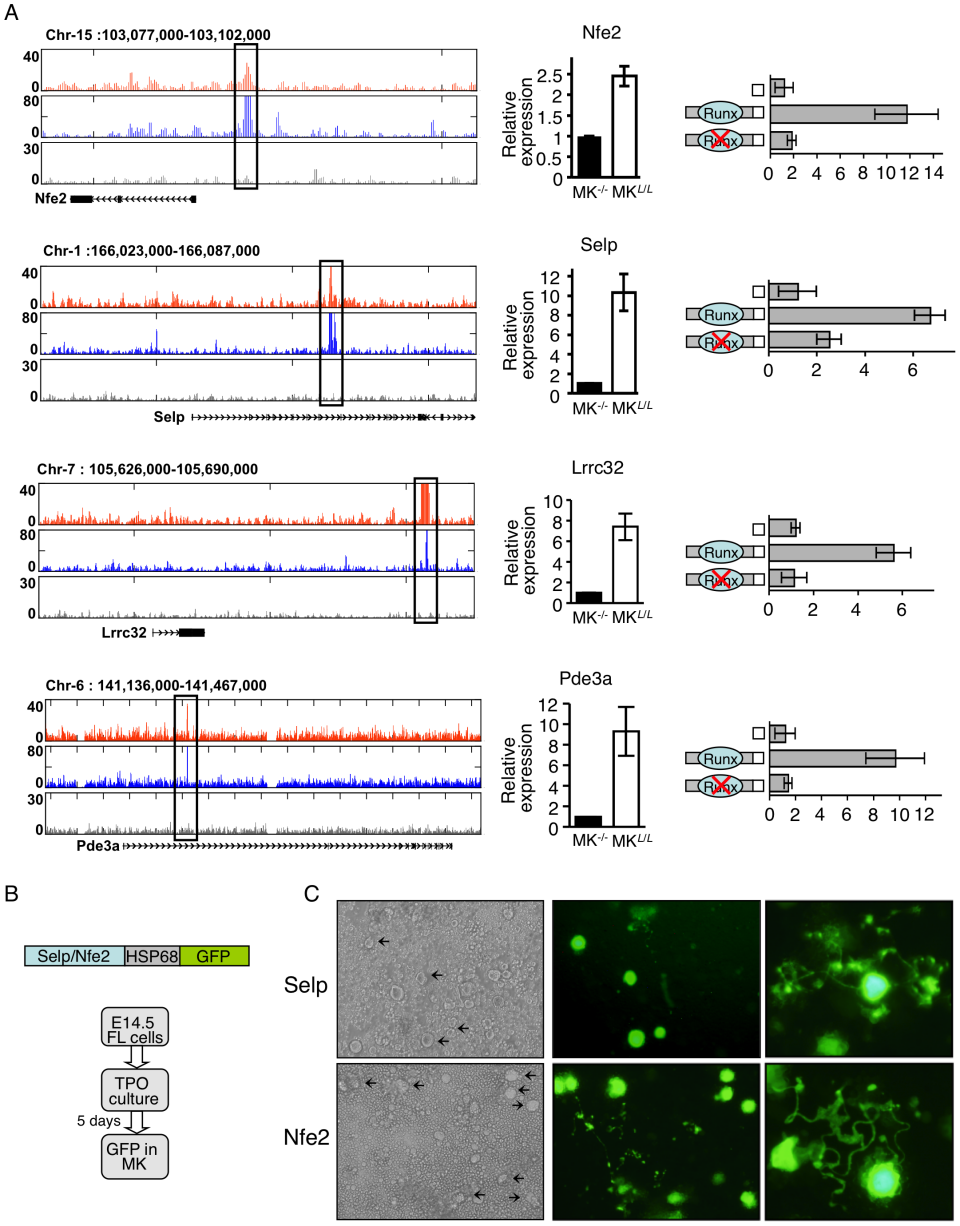
Validation of Runx1/p300 bound megakaryocytic enhancers. (A) Transfection-mutagenesis validation of Runx1 dependent activation of *Nfe2*, *Selp*, *Lrrc32* and *Pde3a* regulatory elements by transfection into megakaryocytic cell line Meg01. Tracks of Runx1 (orange), p300 (blue) and non-immune serum (gray) ChIP-seq traces in maturating FL-MK (left panels). The cloned Runx1/P300 bound regions are marked by quadrangles. RT-qPCR analysis recording expression of the genes in FL-MK^Runx1−/−^ (MK^−/−^), or FL-MK^Runx1*L*/*L*^ (MK*^L^* ^/*L*^) (middle panels). The data represent means ± SD of two experiments performed in triplicates. Dual luciferase reporter assays in transfected Meg01 cells using PGL4.73 vector alone or with the indicated intact/mutated regulatory region. (B) Transgenic analysis of *Selp and Nfe2* regulatory regions. Co-bound Runx1/p300 regulatory regions were cloned upstream of the HSP68 promoter-GFP constructs. FL cells of E14.5 PCR-GFP-positive transgenic embryos were cultured with TPO 50 ng ml^−1^ for 3–5 days before evaluation of GFP expression. (C) Light and fluorescence microscope images of 4-days cultured FL cells derived from E14.5 Selp (15/26 positive embryos) or Nfe2 (11/20 positive embryos) transgenic embryos. Images were produced using an Olympus IX71 microscope with 10×/0.3 (left and middle) and 40×/0.6 (right) objective lenses.

To further evaluate the biological relevance of Runx1/p300 co-bound enhancers we examined four evolutionary conserved co-bound regions located at the vicinity of *Nfe2*, *Selp*, *Lrrc32* and *Pde3a* gene loci (Figure 7A). These four genes, which play key roles in megakaryopoiesis and platelet function [19], [32], [33], [34], [35], [36], are among Runx1 regulated genes (Figure 2F&G and Figure 7A). Genomic regions shown in Figure 7A were cloned into the pGL4 luciferase-SV40-reporter construct and evaluated by ectopic expression. Following transfection into the megakaryoblastic cell line Meg01, the four regions conferred Runx1-dependent activation of the basal promoter activity (Figure 7A). Two of the regions (*Nfe2* and *Selp*) were also cloned into GFP reporter constructs containing the basal promoter of the Hsp68 gene, which by itself is largely silent and does not confer expression in hematopoietic cells [37]. These constructs were microinjected to fertilized eggs and single cell suspensions derived from FL of transgenic E14.5 embryos were cultured with TPO for 3–5 days, following by evaluation of reporter expression (Figure 7B). Significantly, Runx1-bound, *Nfe2* and *Selp* regulatory regions conferred GFP expression specifically in mature, pro-platelet megakaryocytes (Figure 7C), consistence with the role of these genes in late stages of megakaryopoiesis, platelet synthesis and function.

## Discussion

The megakaryopoiesis process leading to production of platelets involves profound cell morphological changes and is transcriptionally regulated at multiple stages. In this study we demonstrated that Runx1 functions as a key regulator in the transcription program driving megakaryocytic maturation and platelet production. This notion is supported by the phenotypic defects of Runx1*^F^* ^/*F*^/Pf4-Cre mice in which Runx1 is inactivated at late stages of megakaryocytic maturation. Genome wide occupancy profile indicated that Runx1 and its transcriptional collaborators, including the co-activator p300, regulate the target genes mostly though binding to distant-acting regulatory elements. Integrated ChIP-seq and differential gene expression analysis identified subset of Runx1 regulated genes bearing functional importance to megakaryocytic maturation and platelet production and provided information about potential cooperating TFs in this process.

### Runx1 mediated transcriptome reflects its crucial role in late stage megakaryopoiesis and platelets production

Analysis of primary FL MK derived from Runx1*^F^* ^/*F*^/Pf4-Cre mice delineated for the first time detailed information about Runx1 mediated expression in advanced stages of megakaryocytic differentiation. To drive this developmental program Runx1 up-regulates genes playing roles in platelets formation and function and represses expression of cell proliferation-inducing genes. Upon loss of Runx1 the program is abrogated leading to aberrant megakaryocytic maturation, sustained proliferation of FL-MK^Runx1−/−^ and thrombocytopenia in Runx1*^F^* ^/*F*^/Pf4-Cre mice. These data underscores the central role of Runx1 in gene expression regulation at late megakaryopoiesis and may provide better understanding of Runx1 associated FPD-AML.

### Characteristics of Runx1 occupancy regions

As noted above, in maturating MK, Runx1-mediated gene expression involved binding of Runx1 to a large number of genomic regions, most of which were remote from TSSs. Interestingly, we found a significant enrichment of Runx1 ChIP-seq peaks within 50 kb surrounding the TSS of up-regulated genes, whereas repressed genes were enriched in regions spanning 70 kb to 130 kb around the TSS. This apparent distance difference between up- or -down regulated genes may suggest that among other parameters the distance of Runx1 bound regions relative to the TSS is contributing to its function in maturating MK.

Sequence analysis revealed that the RUNX motif was highly enriched within the Runx1 bound regions and positively correlated with the Runx1 binding strength as reflected in the number of ChIP-seq reads. Significant correlation was also noted with ETS TF binding motif, which was the second most enriched within the Runx1 bound regions, whereas the GATA motif was enriched to a lesser degree. Using TPA treated K562 cells we have previously found that in early stages of megakaryocytic differentiation the AP-1 TF complexes were the main cooperating partners of RUNX1. Moreover, to drive early differentiation gene expression RUNX1 directly up-regulated the expression of the AP-1 factors FOS, FOSB and JUN, which in turn recruited RUNX1 to *de-novo* binding regions lacking RUNX binding motif [8]. In sharp contrast, in maturating MK Runx1 acts to directly repress Ap-1 and cooperates with Ets TF to drive late stages of MK differentiation and platelets production. This occurrence underscores the dynamics of the megakaryopoietic differentiation program driven by RUNX1 through sequential stage specific cooperation with several collaborating TFs.

### Runx1/p300 co-occupancy in maturating megakaryocytes

Genome wide p300 ChIP-seq in maturating FL-MK yielded thousands of occupied regions predominantly at sites remote from TSS. This pattern corresponds with the previously demonstrated preference of p300 for binding at enhancer regions [17], [38], [39]. More recently, Vahedi et al [40] have demonstrated that p300 binding pattern provides important information for identification of active enhancer regions. Runx1/p300 co-bound regions comprised ∼10% of the Runx1 occupied regions. Nevertheless, this distinct subset of co-bound loci defines a group of genes that are highly important to platelet formation and function. Interestingly, many of these Runx1/p300 co-bound genes did not respond to loss of Runx1. This finding underscores the notion that at a given stage during MK differentiation only a subset of Runx1 bound enhancers is active and the differentiation program proceeds through dynamic interactions of the epigenome with Runx1 and its stage specific collaborating TFs [8]. Thus, not all co-bound genes were differentially expressed in Runx1^−/−^ FL-MK, reflecting the program's dynamics and the above noted stage-heterogeneity of maturating primary FL-MK. Overall, this genome-wide analysis of Runx1/p300 co-bound distant-acting regulatory elements provided important information about the Runx1 mediated transcriptional program during final stages of megakaryocytic maturation.

## Supporting Information

Table S1 **Listed are genes that are >1.8 fold up- or down-regulated in MK^RUNX1−/−^** **relative to MK^RUNX1−^** ^***L*/*L***^ **(** ***P*** **<0.05) and are known to play role in cell proliferation and leukemia, or in megakaryopoiesis and platelet function. PubMed IDs indicating the relevant studies pertained to these genes are indicated.**
(DOC)

Table S2 **listed are the sequence of the various primers used for PCR analyses.**
(DOCX)
