# Supplementary material for: Cell-Autonomous Function of Runx1 Transcriptionally Regulates Mouse Megakaryocytic Maturation
Source: PLoS One. 2013 May 23;8(5):e64248. doi: 10.1371/journal.pone.0064248 (PMC3662678; doi:10.1371/journal.pone.0064248)
Supplement: Table S1 — Listed are genes that are >1.8 fold up- or down-regulated in MKRUNX1−/− relative to MKRUNX1− L/L ( P <0.05) and are known to play role in cell proliferation and leukemia, or in megakaryopoiesis and platelet function. PubMed IDs indicating the relevant studies pertained to these genes are indicated. (DOC) [file pone.0064248.s001.doc]

**Supporting information**

**Table S1.** **Runx1 responding genes that play role in cell proliferation, megakaryopoiesis and platelet function.**

|  | Gene name | **Gene symbol** | **Fold change KO vs. WT** | **P-value** | **Pubmed ID** |
| --- | --- | --- | --- | --- | --- |
| Genes involved in proliferation and leukemia | | | | | |
| 1 | B-cell leukemia/lymphoma 2 | Bcl2 | 1.80972 | 0.002858 | 18838203 |
| 2 | B-cell leukemia/lymphoma 3 | Bcl3 | 6.74507 | 2.04E-05 | 7896265 |
| 3 | Adenovirus E1B interacting protein 3 | Bnip3 | 2.22901 | 0.02616 | 18838203 |
| 4 | Carcinoembryonic antigen-related cell adhesion molecule | Ceacam1 | -3.11296 | 1.28E-05 | 19008452 |
| 5 | Avian erythroblastosis virus E-26 (v-ets) oncogene related | Erg | 1.81411 | 0.003894 | 19487285 |
| 6 | E26 avian leukemia oncogene 1 | Ets1 | 1.91015 | 0.00135 | 15466856 |
| 7 | E26 avian leukemia oncogene 2 | Ets2 | 6.27392 | 0.000384 | 18094917 |
| 8 | FBJ osteosarcoma oncogene | Fos | 1.95135 | 0.01756 | 19381435 |
| 9 | FBJ osteosarcoma oncogene b | Fosb | 1.93859 | 0.016729 | 19381435 |
| 10 | Jun oncogene | Jun | 2.71854 | 0.00421 | 15707584 |
| 11 | Kinase insert domain protein receptor | Kdr | 2.84772 | 0.000319 | 15962006 |
| 12 | Kit oncogene | Kit | 7.44435 | 0.00023 | 16492768 |
| 13 | NAD(P)H dehydrogenase, quinone 1 | Nqo1 | -1.98208 | 0.004606 | 12036909 |
| 14 | Pyruvate dehydrogenase kinase, isoenzyme 1 | Pdk1 | 1.83128 | 0.023778 | 10896934 |
| 15 | Protein tyrosine phosphatase, non-receptor type 11 | Ptpn11 | -1.84749 | 1.81E-05 | 19773259 |
| 16 | BH3 interacting domain death agonist | Bid | -1.90476 | 0.003821 | 18665919 |
| Genes involved in megakaryopoiesis | | | | | |
| 17 | ATPase, Ca++ transporting, plasma membrane 4 | Atp2b4 | -2.49452 | 2.38E-05 | 17883705 |
| 18 | Caspase 12 | Casp12 | -2.4143 | 0.000155 | 15059849 |
| 19 | CD34 antigen | Cd34 | 2.92322 | 2.84E-05 | 19619605 |
| 20 | CD84 antigen | Cd84 | -2.11084 | 4.90E-05 | 16037392 |
| 21 | CD93 antigen | Cd93 | -2.64064 | 0.001159 | 16490927 |
| 22 | Chemokine (C-X-C motif) receptor 4 | Cxcr4 | -2.98799 | 0.004002 | 19006565 |
| 23 | Dual specificity phosphatase 2 | Dusp2 | 2.49279 | 0.018599 | 14717982 |
| 24 | Early growth response 1 | Egr1 | 3.31023 | 0.000834 | 19747485 |
| 25 | Endoglin | Eng | 2.76021 | 0.005055 | 17673527 |
| 26 | Filamin, alpha | Flna | -3.6267 | 0.00085 | 18802008 |
| 27 | FMS-like tyrosine kinase 1 | Flt1 | -2.15274 | 0.007664 | 12406876 |
| 28 | FYN binding protein | Fyb | -2.02546 | 2.23E-05 | 17003372 |
| 29 | Hemogen | Hemgn | -2.61515 | 3.84E-05 | 18599389 |
| 30 | Heme oxygenase (decycling) | Hmox1 | -2.41561 | 0.004233 | 18983509 |
| 31 | Interleukin 6 signal transducer | Il6st | 2.70741 | 0.003892 | 12423677 |
| 32 | Integrin alpha L | Itgal | -4.52159 | 0.000428 | 15693793 |
| 33 | Leukemia inhibitory factor receptor | Lifr | 5.49701 | 0.001362 | 7540557 |
| 34 | Leucine rich repeat containing 32 | Lrrc32 | -6.74817 | 2.66E-06 | 17192395 |
| 35 | V-maf musculoaponeurotic fibrosarcoma oncogene | Mafg | -2.18503 | 0.001093 | 9679061 |
| 36 | Myristoylated alanine rich protein kinase C substrate/ | Marcks | 2.29811 | 0.007778 | 8703801 |
| 37 | MKL (megakaryoblastic leukemia)/myocardin-like 1 | Mkl1 | -1.80233 | 0.021284 | 19136660 |
| 38 | Myosin, light polypeptide 9 | Myl9 | -20.0004 | 2.82E-09 | 19724058 |
| 39 | Nuclear distribution gene C homolog (Aspergillus) | Nudc | 1.86818 | 5.22E-05 | 16440315 |
| 40 | Purinergic receptor P2Y, G-protein coupled 12 | P2ry12 | -2.35573 | 0.000382 | 15914557 |
| 41 | Pre B-cell leukemia transcription factor 1 | Pbx1 | 2.81918 | 0.000109 | 12609849 |
| 42 | Phosphatidylinositol 3-kinase, catalytic, beta polypeptide | Pik3cb | -2.19294 | 1.72E-08 | 18256550 |
| 43 | Phosphoinositide-3-kinase, catalytic, gamma polypeptide/ | Pik3cg | -1.93546 | 5.52E-07 | 17464189 |
| 44 | Phospholipase A2, group IVA (cytosolic, calcium-dependent) | Pla2g4a | -5.78489 | 2.37E-07 | 9879666 |
| 45 | Prostaglandin I receptor (IP) | Ptgir | -5.15523 | 0.000283 | 15326037 |
| 46 | Prostaglandin-endoperoxide synthase 1 | Ptgs1 | -3.65685 | 1.06E-05 | 9022759 |
| 47 | Ras homolog gene family, member C | Rhoc | -2.11357 | 0.000722 | 8608802 |
| 48 | Selectin, platelet | Selp | -14.9784 | 4.02E-09 | 19669980 |
| 49 | Serine (or cysteine) peptidase inhibitor, clade B, member 2 | Serpinb2 | -7.3313 | 0.000112 | 9182567 |
| 50 | Serine (or cysteine) peptidase inhibitor, clade member E 1 | Serpine1 | 1.8994 | 0.010612 | 10397715 |
| 51 | Serine (or cysteine) peptidase inhibitor, clade member E 2 | Serpine2 | -5.97601 | 3.01E-05 | 10397715 |
| 52 | Suppressor of cytokine signaling 3 | Socs3 | 2.67018 | 0.034165 | 19225535 |
| 53 | Signal transducer and activator of transcription 3 | Stat3 | 1.95502 | 2.71E-05 | 18060035 |
| 54 | Thromboxane A2 receptor | Tbxa2r | -2.53598 | 0.005292 | 19747485 |
| 55 | Transforming growth factor, beta 1 | Tgfb1 | -1.91777 | 0.000114 | 16938119 |
| 56 | Transient receptor potential cation channel, subfamily C, member 1 | Trpc1 | -4.33342 | 2.67E-05 | 16857711 |
| 57 | Wiskott-Aldrich syndrome homolog (human) | Was | -1.96048 | 1.71E-05 | 12591280 |
| 58 | Zyxin | Zyx | -2.25464 | 0.000418 | 15120620 |
| Gene involved in platelets function | | | | | |
| 59 | ATP-binding cassette, sub-family A (ABC1), member | Abca1 | 2.03991 | 0.009706 | 16855366 |
| 60 | ATP-binding cassette, sub-family A (ABC1), member 7 | Abca7 | -1.84981 | 0.010396 | 12727224 |
| 61 | Adenosine deaminase | Ada | 1.81801 | 0.000473 | 12893689 |
| 62 | A disintegrin and metallopeptidase domain 10. | Adam10 | -1.99557 | 0.000121 | 17445093 |
| 63 | Alpha fetoprotein | Afp | 1.88089 | 0.032476 | 2468586 |
| 64 | Angiopoietin 1 | Angpt1 | -1.95528 | 0.005387 | 11246533 |
| 65 | Apolipoprotein E | Apoe | 3.41094 | 0.002183 | 19687360 |
| 66 | Rho guanine nucleotide exchange factor (GEF) 12 | Arhgef12 | 2.26301 | 0.001767 | 17074726 |
| 67 | CD28 antigen | Cd28 | -5.88352 | 2.29E-05 | 17096917 |
| 68 | CD59b antigen | Cd59b | -2.2682 | 8.33E-05 | 19229985 |
| 69 | 2',3'-cyclic nucleotide 3' phosphodiesterase | Cnp | -2.00027 | 0.003158 | 16179391 |
| 70 | Collagen, type XVIII, alpha 1 | Col18a1 | 2.29018 | 2.41E-05 | 17883697 |
| 71 | Chemokine (C-X-C motif) ligand 2 | Cxcl2 | 1.90144 | 0.018201 | 10891429 |
| 72 | Docking protein 2 | Dok2 | -6.09043 | 2.77E-08 | 17092301 |
| 73 | Destrin | Dstn | -2.53409 | 0.000206 | 2578390 |
| 74 | Eph receptor A4 | Epha4 | 6.0222 | 1.76E-05 | 15994237 |
| 75 | Fc receptor, IgG, low affinity IIb | Fcgr2b | 4.99092 | 0.012594 | 18565176 |
| 76 | Hermansky-Pudlak syndrome 4 homolog | Hps4 | -1.86756 | 6.51E-05 | 12664304 |
| 77 | Heparanase | Hpse | -13.5392 | 2.79E-06 | 9494105 |
| 78 | Il6ra // interleukin 6 receptor, alpha | Il6ra | -3.96823 | 0.001225 | 9685167 |
| 79 | Integrin linked kinase | Ilk | -1.91915 | 6.64E-05 | 18772455 |
| 80 | Integrin alpha 2 | Itga2 | -9.19719 | 1.91E-05 | 19258597 |
| 81 | Latent transforming growth factor beta binding protein 2 | Ltbp2 | -2.59689 | 0.055988 | 7798248 |
| 82 | Alpha globin regulatory element containing gene | Mare | -1.8046 | 2.75E-05 | 8980258 |
| 83 | 5,10-methylenetetrahydrofolate reductase | Mthfr | 2.78058 | 0.000927 | 16011963 |
| 84 | Myomesin 1 | Myom1 | -3.25626 | 0.000713 | 19117493 |
| 85 | Neuropeptide Y | Npy | -25.7417 | 0.012533 | 7668111 |
| 86 | Neurogranin | Nrgn | -1.89599 | 8.53E-05 | 12433680 |
| 87 | N-ethylmaleimide sensitive fusion protein | Nsf | 2.1357 | 0.000247 | 17065550 |
| 88 | ORAI calcium release-activated calcium modulator 2 | Orai2 | -2.94121 | 0.000118 | 19695217 |
| 89 | Phosphodiesterase 3A, cGMP inhibited | Pde3a | -7.3289 | 1.08E-06 | 19261611 |
| 90 | Phosphoglucomutase 1 | Pgm1 | -3.78041 | 6.73E-07 | 12804634 |
| 91 | Podocalyxin-like | Podxl | 4.89018 | 0.000361 | 17922228 |
| 92 | Prolyl endopeptidase | Prep | -2.1971 | 6.11E-06 | 2295656 |
| 93 | Protein kinase C, delta | Prkcd | -2.00515 | 6.64E-07 | 14578358 |
| 94 | Protein tyrosine phosphatase, non-receptor type 1 | Ptpn1 | -1.97911 | 0.00011 | 16115959 |
| 95 | Protein tyrosine phosphatase, receptor type, J | Ptprj | -2.00912 | 0.000256 | 19246339 |
| 96 | 6-pyruvoyl-tetrahydropterin synthase | Pts | -2.36489 | 0.015856 | 7981621 |
| 97 | RAB27A, member RAS oncogene family | Rab27a | -4.50784 | 7.49E-05 | 17384153 |
| 98 | Regulator of G-protein signaling 14 | Rgs14 | -1.906 | 0.001784 | 17074726 |
| 99 | Serum deprivation response | Sdpr | -1.87394 | 0.001819 | 19852682 |
| 100 | Serine (or cysteine) peptidase inhibitor, clade member B 6a | Serpinb6a | -2.47282 | 0.000217 | 17761692 |
| 101 | Solute carrier family 18 (vesicular monoamine), member 2 a | Slc18a2 | 2.98714 | 0.002465 | 12604601 |
| 102 | Solute carrier family 24 (sodium/potassium/calcium exchanger member a3 | Slc24a3 | -2.56521 | 5.46E-08 | 17059412 |
| 103 | Solute carrier family 6 | Slc6a4 | -3.8124 | 1.38E-05 | 18317590 |
| 104 | Secretory leukocyte peptidase inhibitor | Slpi | 3.64826 | 0.001802 | 15315966 |
| 105 | Stomatin | Stom | -4.54869 | 7.28E-05 | 12130500 |
| 106 | Thrombomodulin | Thbd | -2.44911 | 0.01445 | 12595308 |
| 107 | Tyrosine kinase with immunoglobulin-like and EGF-like domain 1 | Tie1 | -1.85138 | 0.005272 | 11146396 |
| 108 | Translocator protein | Tspo | -1.91702 | 0.003355 | 18054208 |
| 109 | Tubulin, beta 1 | Tubb1 | -1.92155 | 0.000243 | 15956286 |
| 110 | Vasodilator-stimulated phosphoprotein | Vasp | -2.01505 | 0.001224 | 10393958 |
| 111 | Vascular endothelial growth factor C | Vegfc | -2.0635 | 0.000282 | 9684805 |
| 112 | Vimentin | Vim | 2.25522 | 0.000439 | 11744725 |
| 113 | ATP-binding cassette, sub-family A (ABC1), member 1 | Abca1 | 2.03991 | 0.009706 | 16855366 |
