# Supplementary material for: Cell-Autonomous Function of Runx1 Transcriptionally Regulates Mouse Megakaryocytic Maturation
Source: PLoS One. 2013 May 23;8(5):e64248. doi: 10.1371/journal.pone.0064248 (PMC3662678; doi:10.1371/journal.pone.0064248)
Supplement: Table S2 — listed are the sequence of the various primers used for PCR analyses. (DOCX) [file pone.0064248.s002.docx]

**Table S2. Sequence of primers used for PCR analyses**

| **Name** | **Sequence 5’ – 3’** |
| --- | --- |
| PBGD-mouse | Taqman assay Mm00660262 |
| PBGD-mouse | f- ctcaaagatgagggtgattagag |
|  | r- ccgtggtggacatagcaatga |
| Runx1-mouse | Taqman assay Mm01213404 |
| Fos-mouse-RNA | f- ctgacagatacactccaagc |
|  | r- tcagtctgcaacgcagaactt |
| Fosb-mouse-RNA | f- tctcagtacctgtcttcggt |
|  | r- ttgtgattgcggtgaccgtt |
| Jun-mouse-RNA | f- aaagcgcaaaactccaggct |
|  | r- agttggcacccactgttaac |
| Junb-mouse-RNA | f- atgaccttctgcttgagctg |
|  | r- tggaggacaaggtgaagaca |
| Pde3a-mouse-RNA | f- ccattcagaatgggaccaca |
|  | r- aggatgtacacacattcggg |
| Lrrc32-mouse-RNA | f- tatctctcagcgtcgagagc |
|  | r- aggatgctctgcagttggtt |
| Myl9-mouse-RNF | f- tcagaaccgagatggcttca |
|  | r- acatggtgaagttgatgggc |
| Selp-mouse-RNA | f- taagatgcctggctactgga |
|  | r- aattcccaagaggctgaacg |
| Npy-mouse-RNA | f- tgtttgggcattctggctga |
|  | r- ctgaaatcagtgtctcaggg |
| Itga2-mouse-RNA | f- agcagcttacgaacccacaa |
|  | r- aatcccaggctcatgttggt |
| Lifr-mouse-RNA | f- ctggtgatcacgaagtcaca |
|  | r- ctgtcgttccacttcagtag |
| Ets2-mouse-RNA | f- tttgtgggtgacatcctctg |
|  | r- catgctgtccaggagattgt |
| Kit-mouse-RNA | f- aatacgaggcctaccccaaa |
|  | r- ggtataagtgcctccttctg |
| Cd34-mouse-RNA | f- tggaagtaccagccactact |
|  | r- agtagtaggcagtatgccag |
| Nfe2-mouse-RNA | f-gatcatgtccattactgagc |
|  | r- gaagcctgcatctggatgaa |
| Pf4-cre | f- cccatacagcacaccttttg |
|  | r- tgcacagtcagcaggtt |
| Runx1-condotional KO | f- gagtcccagctgtcaattcc |
|  | r- ggtgatggtcagagtgaagc |
| Runx1 KO southern | f- tgcgcttacagaatgtcagg |
|  | r- catgaccatgattgcaggag |
| **ChIP-seq validation primers** | |
| Fos-ChIP-P-mouse | f- cagtgacgtaggaagtccat |
|  | r- gctctatccagtcttctcag |
| Fos-ChIP-E1-mouse | f- tggtgtgcacatcttcctga |
|  | r- gtgtgcattgtttagcctgtg |
| Fos-ChIP-E2-mouse | f- agttgcctcttagttggcct |
|  | r- ggttgaaggtttcttgaggc |
| Fosb-ChIP-P-mouse | f- ccgagctccttatatggcta |
|  | r- tccatgcacaaacctcccaa |
| Fosb-ChIP-E1-mouse | f- actttctcttcctgtcctgc |
|  | r- ctgaaatcagtcaccttggg |
| Fosb-ChIP-E2-mouse | f- actttctcttcctgtcctgc |
|  | r- ctgaaatcagtcaccttggg |
| Jun-ChIP-P-mouse | f- atggagacctcaccctagaa |
|  | r- aagacgtcagcccacaatg |
| Jun-ChIP-E1-mouse | f- ctggttattgtcccaagtcc |
|  | r- ggcaggagaaagactacatg |
| Jun-ChIP-E2-mouse | f- ctggttattgtcccaagtcc |
|  | r- aaggtcagtctatctcgtgc |
| Pde3a-ChIP | f- ggattgccagtgagcttaatg |
|  | r- ctgctgtgggcatgatacaa |
| Lrrc32-ChIP | f- tcatcagaatggtggcttcc |
|  | r- aatggccatctccactcacca |
| **Transgenic construct generation primers** | |
| Pde3a-bgl2-f | tttcttagatctcccagtgtccatcttttctc |
| Pde3a-bgl2-r | attcaaagatctcgtcaatgccttgcttctga |
| Pde3a-mut1 | f- tttaagtctaaaatgtcaagatacttattatgttc |
|  | r- cattttagacttaaaatagaaagttgttaagtaag |
| Pde3a-mut2 | f- cccgagtctaagctttcccacgctccttttttggc |
|  | r- aagcttagactcggggtgaggggttagggaaacag |
| Lrrc32-bgl2-f | acagaaagatctcttctaagtcctaaccacagc |
| Lrrc32-bgl2-r | aagcaaagatctggtctagaaagctatgaggtc |
| Lrrc32-mut | f- gcgtcactttctcactttgcccacaaagcctaaaaa |
|  | r- aaagtgagaaagtgacgcgggggttagcacggcc |
| Selp- Hind3-f | f- acgataaagctttccttcgttcaaggcatcag |
| Selp- Sph1-f | r- gtcagagcatgctcgagagcgaacagattctc |
| Selp-mut | f- cactcattccagcttcctgtgaccttcca |
|  | r- gaagctggaatgagtggcaggccctggca |
| Nfe2-Hind3-f | attttcaagcttcacttttgctgctcacccaa |
| Nfe2-Sph1-f | gtcagagcatgctcgagagcgaacagattctc |
| Nfe2-mut | f- gatcatgtccattactgagc |
|  | r- gaagcctgcatctggatgaa |
